# Antimicrobial Activity of Lipopeptide Biosurfactants Against Foodborne Pathogen and Food Spoilage Microorganisms and Their Cytotoxicity

**DOI:** 10.3389/fmicb.2020.561060

**Published:** 2021-01-11

**Authors:** Konstantina Kourmentza, Xavier Gromada, Nicholas Michael, Charlotte Degraeve, Gaetan Vanier, Rozenn Ravallec, Francois Coutte, Kimon Andreas Karatzas, Paula Jauregi

**Affiliations:** ^1^Department of Food and Nutritional Sciences, School of Chemistry, Food and Pharmacy, University of Reading, Reading, United Kingdom; ^2^UMR Transfrontalière BioEcoAgro No 1158, University Lille, INRAE, University Liège, UPJV, YNCREA, University Artois, University Littoral Côte d’Opale, ICV – Institut Charles Viollette, Lille, France; ^3^Chemical Analysis Facility (CAF), Department of Chemistry, School of Chemistry, Food and Pharmacy, University of Reading, Reading, United Kingdom; ^4^Lipofabrik, Polytech-Lille, Villeneuve d’Ascq, France

**Keywords:** lipopeptides, antimicrobial, mycosubtilin, antifungal, food spoilage, nisin, *B. subtilis*

## Abstract

Lipopeptide biosurfactants produced by *Bacillus* sp. were assessed regarding their antimicrobial activity against foodborne pathogenic and food spoilage microorganisms. Both Gram-positive and Gram-negative bacteria were found not to be susceptible to these lipopeptides. However, mycosubtilin and mycosubtilin/surfactin mixtures were very active against the filamentous fungi *Paecilomyces variotti* and *Byssochlamys fulva*, with minimum inhibitory concentrations (MICs) of 1–16 mg/L. They were also active against *Candida krusei*, MIC = 16–64 mg/L. Moreover it was found that the antifungal activity of these lipopeptides was not affected by differences in isoform composition and/or purity. Furthermore their cytotoxicity tested on two different cell lines mimicking ingestion and detoxification was comparable to those of approved food preservatives such as nisin. Overall, for the first time here mycosubtilin and mycosubtilin/surfactin mixtures were found to have high antifungal activity against food relevant fungi at concentrations lower than their toxicity level hence, suggesting their application for extending the shelf-life of products susceptible to these moulds. In addition combining nisin with mycosubtilin or mycosubtiliin/surfactin mixtures proved to be an effective approach to produce antimicrobials with broader spectrum of action.

## Introduction

According to the Food and Agriculture Organization of the United Nations (FAO), every year around 1.3 billion tonnes of food is lost or wasted around the world, corresponding to 1/3 of all food produced for human consumption. At the same time, it has been reported that one in nine people globally remains malnourished (United Nations, 2019). Food losses and waste (FLW) comes up to around 680 and 310 billion US dollars per year in industrialized and developing countries respectively. Fruits and vegetables, along with roots and tubers, have the highest wastage of any food accounting for up to 45%, meaning that almost half of fruit and vegetable production is wasted, followed by fish and seafood (35%), cereals (30%) and oilseeds, meat and dairy (20%) (FAO, 2020; United Nations, 2019).

Food losses and waste can be prevented “at source,” i.e., by the application of pesticides on agricultural products. However, applied pesticides may enter soil and surface waters and percolate down into groundwater, affecting biodiversity in both terrestrial and aquatic ecosystems. Moreover, pesticide residues in food pose a risk to animals and human health as they enter the food chain (Gonzalez Coloma, 2018). Currently, biopesticides are exploited as sustainable alternatives to reduce the use of their chemical counterparts, meet the demands of the growing population, reduce the impact on soil, air and water and improve animals and human health. Among those, biologically active compounds deriving from fermentation, such as biosurfactants, can be used as the active ingredient in biopesticide and biofungicide formulations.

Another way to tackle the problem of FLW is to extent the shelf life of food. An area currently under investigation is the use of bio-based packaging materials coated with antimicrobial agents to reduce microbiological growth, also known as “active packaging.” Antimicrobial agents that can be used, with some being recently investigated, are essential oils (Atarés and Chiralt, 2016; Cofelice et al., 2019), organic acids (Cruz-Romero et al., 2013), bacteriocins (Meira et al., 2017), natural polymers such as chitosan (Zainal Abidin et al., 2019), and biosurfactants (Magalhães and Nitschke, 2013; Meena and Kanwar, 2015).

Biosurfactants are surface-active-agents, produced mainly by microorganisms as secondary metabolites. Such compounds are amphipathic and can decrease the interfacial tension between two immiscible liquids. As such, these compounds are secreted by microorganisms in order to grow on or take up hydrophobic substances (Kosaric and Vardar-Sukan, 2015; Kourmentza et al., 2018).

Due to their amphiphilic nature, biosurfactants comprise at least one hydrophilic moiety, such as a carbohydrate, peptide, carboxyl group and one hydrophobic moiety, usually a fatty acid or a fat alcohol (Jauregi and Kourmentza, 2019; Kourmentza et al., 2019). Lipopeptide biosurfactants are cyclic structures that consist of hydrophilic peptide sequences, of usually 7 to 10 amino acids long, while their hydrophobic moiety comprises a C_13_–C_18_ fatty acid chain. They are mainly produced by *Bacillus* or *Pseudomonas* species (Beltran-Gracia et al., 2017; Ndlovu et al., 2017). Lipopeptides can be classified into different categories depending on their amino acid cyclic sequence. For example, lipopeptides that are produced by strains of *Bacillus subtilis* fall into the categories of surfactin, iturin and fengycin families that have a defined general structure (Jacques, 2011) (Supplementary Figure S1).

Several studies have focused on the microbial production of lipopeptides, by either wild type or genetically engineered strains of *Bacillus* (Coutte et al., 2017, 2013). It has been reported that lipopeptides produced by *Bacillus* strains exhibit various biological activities, such as anti-fungal, anti-inflammatory, anti-tumoral, anti-viral, and anti-platelet that makes them ideal candidates for their application as therapeutic agents and drug delivery systems (Meena and Kanwar, 2015; Kourmentza et al., 2017; Meena et al., 2019). Therefore, they could potentially outcompete their synthetic counterparts in such high-value applications as they have the advantages of being biologically produced in a sustainable way, using renewable resources, while they expected to be less toxic towards humans and the environment (Kopsahelis et al., 2018). However, there is still insufficient knowledge on how these compounds act depending on their structure. This kind of information would be of significant value and provide insight into designing effective products with targeted properties by regulating their microbial production, depending on their final application.

Despite the fact that several studies have been published on the production of lipopeptides, information regarding their antifungal and antibacterial activity is limited and controversial. This is due to the variety of methodologies being used to determine their biological activities, as well as the different purities of the lipopeptide compounds tested. It has to be noted that, most of the studies make use of the crude biosurfactant obtained after solvent extraction. Those results can be misleading as the biological activity can be attributed to the presence of other non-lipopeptide compounds, or those compounds may act in synergy with lipopeptides, enabling biological activity. Lipopeptides purification is not easy and can be very costly. A green method by membrane ultrafiltration has been developed by the authors for single molecules or mixtures where a maximum purity around 90–95% can be reached particularly with surfactin (Coutte et al., 2017). Indeed, the difficulties are more important with mycosubtilin which interacts with protein as reported in previous work by the authors (Jauregi et al., 2013). High purity can be obtained using preparative chromatography but would increase the processing cost drastically particularly, at larger scale.

The aim of this study was to evaluate whether lipopeptides can be used as food preservatives, extending the shelf life and quality of food products by inhibiting microbiological growth. Therefore, for the first time here the antimicrobial activity of lipopeptide biosurfactants produced by *B. subtilis* strains, namely surfactin, fengycin, mycosubtilin, and their mixtures at different purities, were tested against foodborne pathogen and food spoilage microorganisms. In addition, their cytotoxicity was tested on two different cell lines, namely human gut epithelial cells (Caco-2 cells) and a cell line already recommended by EFSA to study the impact of the *Bacillus* sp. metabolites (Vero SF cells from kidney epithelial cells of monkey and evaluate their potential use in food applications. Antimicrobial activity and cytotoxicity of lipopeptides were contrasted with those of food grade preservatives, ethylenediaminetetraacetic acid (EDTA) (E385) and nisin (E234), a polycyclic peptide produced by *Lactococcus lactis* used in preservation of meat, cheese and beverages which has also been incorporated in food packaging. Moreover, samples produced at laboratory scale and semi-industrial scale of different purity will be tested in order to determine the effect of purity on activity.

## Materials and Methods

### Production and Purification of Lipopeptides

Surfactin and fengycin lipopeptides were produced in shake flasks (160 rpm) using Landy media, as previously reported (Coutte et al., 2010a). Briefly, surfactin was produced by the overproducing *B. subtilis* strain BBG131 using Landy medium at 37°C, pH 7.0 during 48 h. This medium was supplemented by 16 mg/L of tryptophan and 0.1 M of MOPS to control the pH during the culture (Coutte et al., 2010b). Fengycin was produced by the *B. subtilis* strain Bs2504 (Ongena et al., 2007) using the same conditions of surfactin production, expect that the temperature was 30°C and the culture time was 72 h. In these culture conditions the strains BBG131 and Bs2504 were able to produce 1.305 ± 0.122 g/L of surfactin and 0.256 ± 0.032 g/L of fengycin, respectively. Broth was then centrifugated for 30 min at 8,000 *g* and the lipopeptides were purified from the supernatant by two steps ultrafiltration methods on 10 kDa membrane (using ethanol as the solvent for the second step of ultrafiltration) including four steps of diafiltration, as previously described by Jauregi et al. (2013). After ethanol evaporation, lipopeptides were freeze dried to obtain powder. The yield of this ultrafiltration purification process at labscale was close to 90%.

**FIGURE 1:**
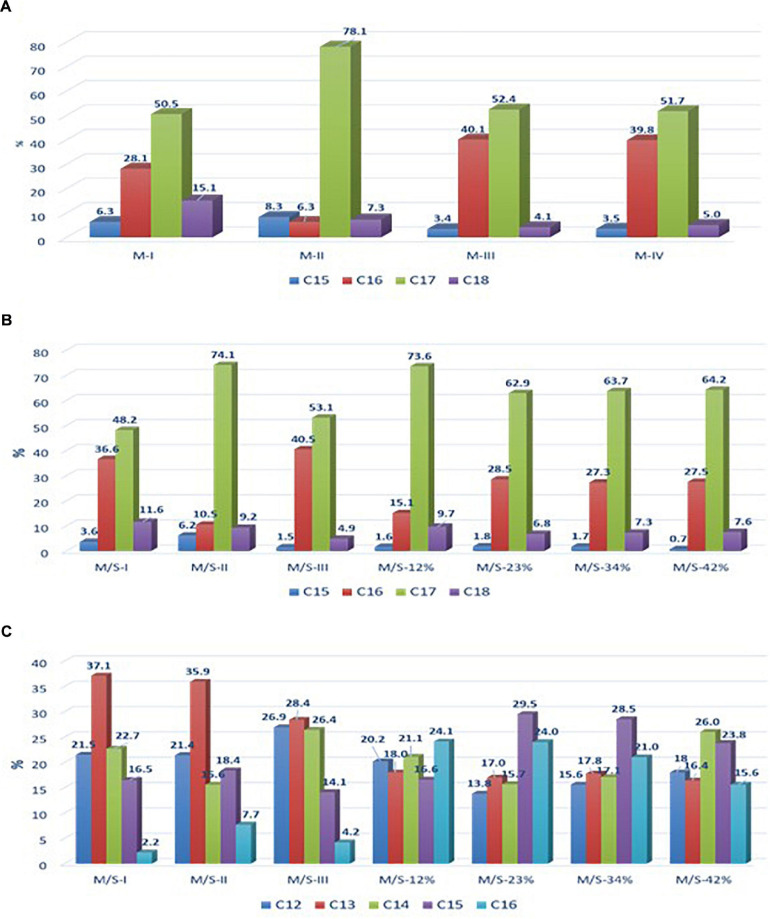
Relative abundance of **(A)** mycosubtilin isoforms in different mycosubtilin samples, **(B)** of mycosubtilin isoforms, and **(C)** surfactin isoforms in different M/S samples.

Mycosubtilin and mixtures of mycosubtilin/surfactin powders were produced and purified on demand by Lipofabrik (Villeneuve d’Ascq, France). Mycosubtilin and the mycosubtilin/surfactin mixtures were produced from two different strains and process at a semi-industrial scale by Lipofabrik (a company specialized in the production of lipopeptides). Mycosubtilin was produced by the strain *B. subtilis* LBS1 and purified. The mixture of mycosubtilin/surfactin (80:20) was produced by the strain *B. subtilis* BLIP2. In both case batch cultures were done in 300 L bioreactor (designed by Lebas Industries, Lezennes, France) containing 200 L of culture media at temperature of 30°C during 72 h and with a pH of 7.0 regulated by adding concentrated solution of NaOH and H_3_PO_4_. After the culture the produced lipopeptides are purified using confidential processes. Solutions containing mycosubtilin or mycosubtilin/surfactin mixtures are extracted at different steps of the purification processes and then freeze dried to obtain powders. The composition is given in Figure 1C.

In these culture conditions the strain BLIP2 was able to produce 0.934 g/L ± 0.045 g/L of lipopeptides with a ratio of 80% of mycosubtilin and 20% of surfactin. The strain LBS1 was able to produce 0.754 g/L ± 0.113 g/L of mycosubtilin.

### Lipopeptides and Other Food Preservatives

The lipopeptides used in this study were surfactin (S), fengycin (F) and mycosubtilin (M) alone and mixtures of surfactin/fengycin (S/F) and mycosubtilin/surfactin (M/S). In addition, in order to evaluate the impact of lipopeptides purity and composition on the antimicrobial activities, mycosubtilin as well as M/S samples were produced and purified on demand by Lipofabrik to obtain variation in purity and in composition (as shown in Table 2).

Nisin (2.5%, Sigma-Aldrich, Gillingham, United Kingdom) and EDTA alone (BioReagent, Sigma-Aldrich) were used as reference food preservatives for antibacterial susceptibility testing, while amphotericin B (European Pharmacopoeia Reference Standard, Sigma-Aldrich) was used as quality control for antifungal susceptibility testing against yeasts and filamentous fungi.

### Characterization of Lipopeptides by Liquid Chromatography Electrospray Ionization Tandem Mass Spectrometry (LC-MS/MS) Analysis

All reagents were LCMS grade and from Fisher Scientific, Loughborough, United Kingdom. Samples were dissolved in 30% acetonitrile/70% water to 50 μg/mL and 2, 5, and 10 μL injects were performed (the inject volumes that gave the greatest signal to noise were analyzed) and separated on a 50 × 2.1 Hypersil Gold C18, 1.9 μM, 100 Å column (Thermo Scientific) using an Accela HPLC system (Thermo Scientific). The mobile phases were (A) water and (B) acetonitrile, both containing 0.1% formic acid. The flow rate was 200 μL/min. The gradient was as follows; 0 min 40% B, 3 min 40% B, 5 min 50% B, 18 min 95% B, 19 min 95% B, 19.5 min 40% B, 25 min 40% B. The outlet went to a UV detector measuring absorbance at 214, 254, and 280 nm and from there into an LTQ-Orbitrap XL mass spectrometer (Thermo Scientific) operating in positive ion mode. Scan event 1 was a full scan at 100,000 resolution in the range 200–2,000 m/z. Scan event 2 fragmented Ions of significant intensity within the LTQ ion trap and then passed them into the Orbitrap for measurement at 15,000 resolution. Phthalate (413.266230 m/z) was used as an internal lock-mass.

Theoretical masses of mycosubtilin and surfactin isoforms were calculated and extracted ion chromatograms for these were generated accordingly. The relative areas of these isoforms in each sample were apportioned as a % and comparisons were made between samples. It has to be noted that this approach does provide absolute quantitation, as different isoforms may ionize with different efficiencies and the lack of pure isolated standards makes a standard curve generation impossible. Nonetheless, this approach was used for performing a relative comparison between samples of relative amounts of isoforms within each sample.

Fengycin was produced by *Bacillus* strain Bs2504 as in our previous work (Hamley et al., 2013) where results of MS analysis can be found.

### Antibacterial Susceptibility Testing of Lipopeptides

The antibacterial susceptibility testing was performed following the broth microdilution methodology, as described previously (Schwalbe et al., 2007), against both Gram-negative and Gram-positive foodborne pathogens and spoilage bacteria. The bacteria used were: *Pseudomonas aeruginosa* NCTC 10332, *Salmonella enterica* NCTC 5188, *Escherichia coli* K-12, *Bacillus cereus* MR59, *Listeria monocytogenes* 10403S, *Carnobacterium divergens* NCFB 2763, *Leuconostoc mesenteroides* NCIMB 08023, and *Brochothrix thermosphacta* NCDO 1676. Moreover, for comparison, along with lipopeptides, nisin and EDTA were also tested. Nisin is a polycyclic antibacterial peptide produced by *Lactococcus lactis* and is an approved food preservative while EDTA is used for the control of microorganisms and biofilms (Finnegan and Percival, 2015; Vessoni Penna et al., 2005).

Since purified lipopeptides and lipopeptide mixtures have low solubility in water they were dissolved in 1% dimethyl sulfoxide (DMSO) and further diluted in the test medium Muller-Hinton (MHB), or Tryptone Soy Broth (TSB) which was alternatively used for *C. divergens*, *L. monocytogenes*, *L. mesenteroides*, and *B. thermosphacta*. Two-fold dilutions were performed and 100 μL of each dilution was placed in each column of a flat bottomed 96-well plate. One column of the plate contained only the growth medium and served as the positive control while in another column contained 200 μL of the growth medium with the highest concentration of the lipopeptide tested (1,000 mg/L) and served as the negative control. Apart from the column that served as the negative control, the rest of the wells were inoculated with 100 μL of the bacterial culture tested. For the bacterial suspension preparation, cultures were incubated overnight in the appropriate medium (MHB or TSB). Bacterial density was adjusted to 0.5 McFarland and suspensions were subsequently diluted 1000 times using the growth test medium. The 96-well plates were then incubated at either 30or 37°C, depending on the optimal growth temperature of each microorganism, for 24 h and the OD_620nm_ was assessed. The minimum inhibitory concentration (MIC) was defined as the lowest concentration of the antimicrobial agent tested that prevented any discernible growth after 24 h incubation.

For testing the antibacterial activity by combining nisin and lipopeptides, the broth microdilution assay described above was used, following the broth microdilution checkerboard panel (Schwalbe et al., 2007).

### Antifungal Susceptibility Testing of Lipopeptides Against Yeasts

The antifungal susceptibility test against yeasts was performed according to the broth microdilution method, as described previously (Urbañski et al., 2002; Schwalbe et al., 2007). *Candida krusei* ATCC 6258 was used as the quality control organism, to verify that antifungal concentrations were prepared properly. In addition, amphotericin B, an effective antifungal antibiotic produced by *Streptomyces nodosus* and part of the WHO List (World Health Organization) of Essential Medicines, was used as a reference compound. The test medium used was RPMI-1640 (with glutamine, without bicarbonate and with a pH indicator) broth with [3-(*N*-morpholino)]-propanesulfonic acid (MOPS) buffer. All tests were performed in U-shaped 96-well plates. Three technical replicates of each assay were carried out on the same day with the same culture and chemical compound with the aforementioned experiment repeated also in triplicate on different days using different cultures (biological replicates) resulting in a total of nine replicates for each assay. The yeasts tested were *Zygosaccharomyces bailli* NCYC 464 and *Debaryomyces hansenii* NCYC 102.

However, due to poor growth of the selected yeasts in the RPMI-1640 medium agar, dilution susceptibility testing was also performed as previously described (Schwalbe et al., 2007) with some modifications. Malt extract agar (MEA) was used as growth medium supplemented with lipopeptides at concentrations ranging from 2 to 264 mg/L. Ten μl of yeast inocula, with 10^5^–10^2^ CFU/mL, were placed on the agar surface and their growth was monitored after 24 and 48 h of incubation at 30°C.

### Antifungal Susceptibility Testing of Lipopeptides Against Filamentous Fungi

The antifungal susceptibility testing of lipopeptides against filamentous fungi was performed according to the broth microdilution methodology, as described previously (Schwalbe et al., 2007). In this set of experiments, *Candida krusei* ATCC 6258 and amphotericin B were used to perform quality control tests for validation. As mentioned above, the test medium used was RPMI-1640 broth with MOPS buffer. Tests were performed in U-shaped 96-well plates in triplicates and the OD_450__nm_ was recorded after 7 days of incubation at 30°C. The fungi tested were *Rhizopus stolonifer* IMI 017314 and *Paecilomyces variotii* and *Byssochamys fulva* IMI 040021.

### Cytotoxity Assays

Cytotoxicity studies were carried-out with the three lipopeptides alone and mixtures of them with nisin and EDTA as food preservative controls on two different cell lines, the Caco-2 cells (human epithelial cells from gut) and the Vero SF cells (monkey epithelial cells from kidney). The Vero SF cells line is recommended by EFSA to assess the toxigenic potential of *Bacillus* species used in animal nutrition (EFSA, 2014). Cells were cultured in Dulbecco’s modified Eagle’s medium supplemented with 10% FCS, 1% glutamine, and 1% penicillin-streptomycin. After 48 h, cell culture medium was replaced by fresh medium containing the tested compounds in various concentrations.

Cytotoxicity was determined through a commercial kit from SIGMA Aldrich (Saint-Louis, United States) named Cell Counting kit 8 (CCK8). This test is based on the water-soluble tetrazolium salt (WST-8) reduction by the dehydrogenase of the living cells only inducing the production of a yellow color formazan dye which is directly proportional to the number of cells. Twenty-four hours after addition the compounds, the WST-8 was added to the medium (100 μL/mL). After 2h incubation, absorption at 650 nm was measured in a microplate-reader (Microplates reader SpectraMax^®^ iD3, Molecular Devices, San Jose, United States) and values were expressed as percent of control. The half maximal inhibitory concentration (IC_50_) was been determined, in agreement with the FDA standard, as the potency of a compound to inhibit 50% of the cell growth.

As explained above, since some of the lipopeptides have limited solubility in aqueous solutions, prior to their cytotoxicity testing, the effect of the DMSO at the levels used to solubilize the lipopeptides, was assessed. DMSO at high concentrations is known to inhibit cell growth, therefore, the DMSO tolerance of each cell line was examined in order to determine a maximum final percentage of 10% DMSO in the culture medium (data not shown).

Each compound was solubilised in a mix of water/DMSO 5% at different concentrations ranging from 2 mg/L to 5 g/L. The compounds tested were mycosubtilin (M-I), surfactin and fengycin as well as different mixtures such as mycosubtilin/surfactin (M/S-I), fengycin/surfactin. Water and DMSO 5% were used as a control. Nisin and EDTA were used between 100 mg/L and 10 g/L.

### Statistical Analysis

For the antimicrobial (antibacterial and antifungal) tests three technical replicates of each assay were carried out on the same day with the same culture and chemical compound with the aforementioned experiment repeated also in triplicate on different days using different cultures (biological replicates) resulting in a total of nine replicates for each assay. Results are reported normally as range of the multiple MIC determinations while when all determinations showed one MIC, this value is reported. The upper and lower values denote the limits all values fall within.

For the cytotoxicity study six technical replicates for each concentration were carried out in the same experiment which was replicated three times. Means values of all the replicates and standard deviation are presented while a Student test was applied to identify statistically significant differences with nisin used as reference.

## Results and Discussion

All MICs results were highly reproducible with all nine replicates giving the same value within a range of two successive dilutions.

### Antibacterial Activity of Food Preservatives

As mentioned above, tests were performed in order to examine the antibacterial activity of nisin (E234) and EDTA (E385) on the selected bacterial strains, which are associated with food spoilage. According to the results obtained, as shown in Table 1, the maximum concentrations of nisin and EDTA used were up to 107.3 mg/L (32 μM) and 29.2 g/L (100 mM) respectively. Nisin alone was only effective against Gram-positive bacteria with MICs ranging between 0.1 and 6.7 mg/L. Among Gram-positive bacteria, *B. thermosphacta* was the most susceptible to nisin (MIC = 0.10–0.20 mg/L) followed by *L. mesenteroides* (MIC = 1.68 mg/L), *C. divergens* (MIC = 3.35 mg/L), *B. cereus* and *L. monocytogenes* (MIC = 6.71 mg/L). These results are in agreement with the literature, since nisin is mainly active against Gram-positive bacteria at concentrations ranging between 1 and 25 mg/L (Zhou et al., 2014, 2016). Nisin has relatively low activity against Gram-negative bacteria since it has difficulty in penetrating the outer membrane that acts as a barrier to its action on the cytoplasmic membrane, which is its main target (Li et al., 2018). Due to the presence of magnesium ions, which play a role in the stabilization of the lipopolysaccharide layer in the outer membrane, chelating agents such as EDTA can facilitate nisin’s action by binding to magnesium ions in the lipopolysaccharide layer and in this way increase susceptibility to antibiotics and detergents (Lambert et al., 2004).

**TABLE 1:** MICs obtained for nisin and EDTA against the bacterial strains studied.

Bacterial strain	Nisin (mg/L)	EDTA (mg/L)
**Gram-negative bacteria**		
*P. aeruginosa* NTCT 10332	>107.3	7,306
*S. enterica* NCTC 5188	>107.3	14,612
*E. coli* K-12	>107.3	14,612
**Gram-positive bacteria**		
*L. monocytogenes* 10403S	6.71	227.94
*B. cereus* MR59	6.71	227.94
*C. divergens* NCFB 2763	3.35	55.58
*L. mesenteroides*	1.68	114.01
*B. thermosphacta*	0.20–0.10	114.01

When EDTA was used on the selected bacterial strains, the MICs for Gram-positive bacteria ranged between 0.19 and 0.78 mM (55.58–227.94 mg/L) while these for Gram-negative bacteria were much higher, ranging between 25 and 50 mM (7,306–14,612 mg/L). EDTA is normally used as a preservative in ocular preparations and eye drops and also as a slime dispersant as it inhibits adherence of strains in intraocular lenses (Kadry et al., 2009). As mentioned above, EDTA is a chelating agent used to destabilize the outer membrane of Gram-negative bacteria allowing antimicrobial agents, such as nisin, quaternary ammonium surfactants, oxacillin, cefamandole, etc., to be more efficient inhibiting their growth (Field et al., 2017; Li et al., 2018).

### Antibacterial Activity of Lipopeptides

Since some lipopeptides have limited solubility in aqueous solutions, prior to their antibacterial testing the effect of the DMSO at the levels used to solubilize the lipopeptides, was assessed. DMSO at high concentrations is known to inhibit microbial growth, therefore, the DMSO tolerance of each strain was examined. The maximum concentration tested was up to 8% DMSO and the growth of the bacterial strains was monitored by checking the OD_620nm_ of the bacterial suspension after 24 h. Our results indicated that DMSO concentrations below 1%, had no inhibitory effect on the bacterial growth of all strains tested. The maximum concentration of lipopeptides tested was up to 1,000 mg/L in the presence of 1% DMSO.

Most of the lipopeptides had no effect on the growth of the bacteria tested (i.e., *P. aeruginosa*, *S. enterica*, *E. coli*, *L. monocytogenes*, *B. cereus*, and *L. mesenteroides*). However, unexpected results were obtained for the Gram-positive bacteria *C. divergens* and in particular, *B. thermosphacta*, with some samples (batches) of mycosubtilin and mycosubtilin/surfactin mixture (Table 2). MICs for *C. divergens* were found to be 500 mg/L for mycosubtilin I (M-I) and mycosubtilin/surfactin I (M/S-I). The same samples were active against *B. thermosphacta*, as MICs of 15.6 and 62.5 mg/L were obtained for M-I and M/S-I respectively. Since surfactin alone did not show any antimicrobial activity the activity of the mixture M/S was attributed to mycosubtilin. However, the MIC values of the latter are extremely high even in comparison with other natural antibacterial molecule such as nisin which inhibits the growth of *B. thermosphacta* and *C. divergens* with a MIC of 0.15 and 3.35 mg/L, respectively (Table 1). These values are 100 to 200 fold lower than those obtained with sample containing mycosubtilin.

**TABLE 2:** MICs of lipopeptides against the Gram-positive bacteria *C. divergens* and *B. thermosphacta.*

Lipopeptide	Composition	Purity	MIC (mg/L)	
	(%)	(%)	*C. divergens*	*B. thermosphacta*
Surfactin (S)		92	>1,000	>1,000
Fengycin (F)		84	>1,000	>1,000
Mycosubtilin I (M-I)		81	500	15.6
Surfactin/Fengycin (S/F)	46:54	72	>1,000	>1,000
Mycosubtilin/Surfactin I (M/S-I)	80:20	>80	500	62.5
Mycosubtilin II (M-II)		>80	>1,000	125
Mycosubtilin III (M-III)		>80	>1,000	>1,000
Mycosubtilin IV (M-IV)		89	>1,000	>1,000
Mycosubtilin/surfactin II (M/S-II)	80:20	>80	>1,000	125
Mycosubtilin/surfactin III (M/S-III)	80:20	61	>1,000	>1,000
Mycosubtilin/surfactin (M/S-23%)	80:20	23	>1,000	15.6
Mycosubtilin/surfactin (M/S-34%)	80:20	34	>1,000	>1,000
Mycosubtilin/surfactin (M/S-42%)	80:20	42	>1,000	>1,000

*M-I, Nov17; M-II, March18; M-III, July18; M-IV, Oct18; M/S-I, Nov17; M/S-II, March18; M/S-III, Oct18.*

So, in order to understand what could lead to the variation in the antibacterial activity of mycosubtilin and mycosubtilin/surfactin samples from different batches two hypothesis were drawn: (i) there is a correlation between mycosubtilin’s isoforms composition and its activity against these two bacteria (ii) the antibacterial activity is exerted by other antibacterial compounds in the samples which are coproduced with lipopeptides by *B. subtilis*.

In order to test the first hypothesis, the isoform composition of mycosubtilin and mycosubtilin/surfactin samples were compared. The LC/MS analysis (Figure 1) show that mycosubtilin isoforms consisted of fatty acid chains of 15 to 18 carbon atoms (C15–C18) (see Supplementary Table S1 for detailed composition data). The relative abundance of isoforms was similar for samples M-III and M-IV and none of these samples showed antibacterial activity (Figure 1A). On the other hand, samples M-I and M-II had different isoform composition and were particularly active against *B. thermosphacta*. In particular, M-II had the highest proportion of C17 isoform (78%) of all samples, but it was at similar proportion in M-I as in M-III and M-IV. Furthermore, M-I had the highest proportion of C18 isoform of all samples (15%). Moreover, the comparison of isoform composition of mycosubtilin in M/S mixtures (Figure 1B) showed that MS-I and M/S-II presented the highest proportion of C18 (≥10%) and C17 (≥74%) isoforms respectively, and display activity (MIC = 15.6 and 125 mg/L, respectively) against *B. thermosphacta*. Nevertheless, M/S III, M/S-34% and M/S-42% which had similar profile of C17 isoform and C18 isoform were not active against *B. thermosphacta*.

A few studies have examined the antibacterial activity of purified lipopeptide homologues against microorganisms and showed that MICs obtained were different as per homologue. For example, C14, C15, and C16 iturin had an MIC of 60, 30, and 7.5 mg/L, respectively, against *Candida albicans*. For *Micrococcus flavus* and *Escherichia coli*, iturin homologues had no effect on their growth. On the other hand, surfactin homologues C14, C15, and C16 showed MICs of 30, 15, and 1.88 mg/L, respectively. In addition, C15 and C16 homologues showed MICs of 60 and 3.75 mg/L, respectively against *E. coli* (Dhanarajan et al., 2016).

From the results of the present study, it was concluded that despite of the differences in the relative abundance of the isoforms there was no conclusive evidence correlating the antibacterial activity with the composition of individual isoforms. So, we could reject the first hypothesis. Another hypothesis and plausible explanation for the variation in activity between different batches was that the antimicrobial activity might be attributed to impurities present in certain samples which, accounted for more than 10 % in each mycosubtilin sample. For example, it has been reported that genes for antimicrobial dipeptides, such as bacilysin, as well as genes for another lantibiotic very active against gram positive bacteria, subtilin and subtilosin A, were present in 93% of the strains (Harwood et al., 2018). Moreover, it is known that the strains used in this study have the ability to produce these antibacterial molecules (Parisot et al., 2008; Fickers, 2012; Velho et al., 2013). The main problem with these peptides is their low stability, in fact they can degrade quickly in the culture medium of *B. subtilis* or during the purification process (Kuboi et al., 1994). This may therefore explain their presence or not in certain of our samples with low purity. Interestingly, this activity was only observed in the batch samples of mycosubtilin (or mycosubtilin/surfactin) produced and purified until March 2018 and not after this date, this period coincides with a change in the purification process of mycosubtilin (confidential data). So, the hypothesis of active impurities purified at the same time as lipopeptides seems to make sense. This theory is supported by the result obtained with high resolution mass spectrometry analysis performed with the sample M-I, using the methodology described by Omoboye et al. (2019) and presented in Supplementary Figure S2. This analysis reveals the presence of two peaks, one at m/z = 830 and another one at 1,107. The one at 1,107 corresponds to mycosubtilin C17 which is the more abundant isoform in this sample (Figure 1) and the one at 830 correspond to the quadricharged ions of subtilin, which gave a total mass of 3,320. All of these results allow us to conclude that the antibacterial activity observed against *B. thermosphacta* is absolutely not due to lipopeptides but rather to the presence of another compound such as subtilin.

Similarly, in the literature when the antibacterial activity of crude lipopeptides is reported other bioactive compounds may be responsible for this activity and it cannot be directly attributed to the presence of lipopeptides.

### Antifungal Activity of Lipopeptides Against Yeasts

In these tests, lipopeptides activity was investigated against the food spoilage relevant yeasts *C. krusei*, *D. hansenii*, and *Z. bailii*. For these tests, the yeast *C. krusei* was used as the reference strain and amphotericin B was used as positive control. According to the obtained results for tests performed in liquid media, mycosubtilin and the M/S samples showed antifungal activity with MICs of 16 and 32–64 mg/L, respectively, after 24 h of growth (Supplementary Table S2). The MIC of mycosubtilin or M/S samples remained similar after 48 h of growth except for the sample M-I, whose MIC increased to 64 mg/L after 48 h. Overall these results show consistent anti-yeast activity as no variation between batches (with different isoform composition) was observed in this case.

Due to poor growth of the yeasts *D. hansenii* and *Z. bailii* in liquid media, susceptibility tests were performed using MEA agar plates, where lipopeptides were incorporated. The growth of all yeasts, including *C. krusei*, was monitored for different cell concentrations (10^2^–10^5^) and MICs were recorded after 24 h of growth for *C. krusei* and *D. hansenii* and after 48 h for *Z. bailii*, as it is a slower growing yeast (Table 3). In agreement with results obtained by the microdilution method (Table 3) only mycosubtilin and the M/S samples showed antifungal activity and similar MIC values were obtained within the range of 8–32 mg/L (Table 3). No MIC was observed for M/S-III, but this sample was active against the yeast *D. hansenii* (MIC = 128 mg/L). All the M/S mixtures were active against the *D. hansenii* with the same MIC (128 mg/L), whereas all samples but MS-III were active against *Z. bailli* with a MIC of 8 mg/L.

**TABLE 3:** MICs (mg/L) obtained for lipopeptides against yeasts grown (at 10^2^–10^3^ CFU/mL) on malt extract agar.

Lipopeptide	Composition	Purity	C. krusei*	Z. bailii**	D. hansenii*
	(%)	(%)			
Surfactin (S)		92	>256	> 256	> 256
Fengycin (F)		84	>256	> 256	> 256
Surfactin/Fengycin (S/F)	46:54	72	>256	> 256	> 256
Mycosubtilin III (M-III)		>80	32	> 256	>256
Mycosubtilin/Surfactin (M/S-I)	80:20	>80	16	8	128
Mycosubtilin/surfactin II (M/S-II)	80:20	>80	8	8	128
Mycosubtilin/surfactin III (M/S-III)	80:20	61	>256	> 256	128

*MIC recorded after 24 h of growth. **MIC recorded after 48 h of growth.

Surfactin is known to have a concentration-dependent mode of action, inducing limited perturbation, such as pore formation, at low concentrations (Heerklotz and Seelig, 2007). In all experiments performed in this study, no antimicrobial activity was observed when surfactin was individually used at concentrations up to 1000 mg/L whilst mycosubtilin was very active against *C. krusei*. However, the M/S mixture was slightly more active against *C. krusei* and only the M/S mixture showed activity against the other two yeasts. Synergistic activities of M/S samples have been already reported for phytopathogenic fungi, such as *F. oxysporum* (Mihalache et al., 2017) and *B. lactucae* (Deravel et al., 2014), and it is also known that anionic and non-ionic biosurfactants, when combined, form mixed micellar structures that act more efficiently on biological membranes than simple micelles (Jauregi et al., 2013; Deravel et al., 2014).

In general, mycosbtilin is characterized by strong antifungal activity against yeasts such as *Candida albicans*, *Candida tropicalis*, *Candida glabrata*, *Cryptococcus neoformans*, *Pichia pastoris*, *Saccharomyces cerevisiae*, and *Yarrowia lipolytica*, among others (Nasir and Besson, 2012). Mycosubtilin belongs to the iturin family, and these biosurfactants are known to increase membrane cell permeability due to the formation of ion-conducting pores, with their characteristics depending on lipid membrane composition and the peptide cycle structure. It has been suggested that ionic pores are attributed to lipopeptide or lipopeptide/phospholipid complex aggregates in the phospholipid membrane (Maget-Dana and Peypoux, 1994; Desmyttere et al., 2019). A study investigating *in vitro* susceptibilities of the abovementioned yeasts to purified anteiso-C17 mycosybtilin homologue showed MIC ranging between 2 and 150 mg/L (Fickers et al., 2009), which are within the range of MICs observed in this study. This is the first time that the activity of lipopeptides has been tested against *C. krusei*, a species involved in chocolate production and an emerging fungal nosocomial pathogen for patients with hematologic malignancies and for transplant recipients, which served also as the quality control. Moreover, lipopeptide susceptibility test against *Z. bailli* and *D. hansenii* were reported here for the first time. *Z. bailii* is one of the most troublesome species for the food industry since spoilage occurring from its growth is quite widespread due to its tolerance to various stress conditions. It is an effective spoiler of several foods and beverages with low pH and high sugar content, such as fruit concentrates, wines, soft drinks, syrups, etc. causing significant economic losses to the food industry (Kuanyshev et al., 2017). On the other hand, *Debaryomyces hansenii*, is an osmo-, halo- and xerotolerant species, common in all types of cheese and the most common yeast in unsulfited and sulfited sausages, skinless sausages and minced beef (Breuer and Harms, 2006). The fact that those species are susceptible to mycosubtilin and M/S lipopeptides enables the use of these lipopeptides as sustainable and low toxicity antifungal agents in food-related and biomedical applications, to restrict the growth of specific fungal pathogens.

### Antifungal Activity of Lipopeptides Against Filamentous Fungi

In this study lipopeptides were tested against food spoilage/pathogen relevant filamentous fungi: (i) *Rhizopus stolonifer* which is commonly known as black bread mold, (ii) *Paecilomyces* variotii, a heat-resistant fungi and common contaminant in heat treated foods and juices, and (iii) *Byssocchlamys fulva*, which is a soil plant pathogen affecting strawberries, pineapples and other fruits and can be also found in pasteurized juices.

According to the results obtained, *R. stolonifer* was not susceptible to any of the lipopeptides tested, at concentrations up to 64 mg/L (Table 4). This is in accordance with the literature, as it has been reported recently that the growth of *R. stolonifer* is only inhibited at higher concentrations of iturin (100 mg/L), fengycin (150 mg/L), and surfactin (200 mg/L) (Liu et al., 2014). On the other hand, *P. variotii* and *B. fulva* were susceptible to all mycosubtilin and M/S samples tested. MICs obtained for any of the mycosubtilin samples were at 2 mg/L and MICs for M/S samples of high purity (>80%) ranged between 1 and 2 mg/L, whereas M/S samples of lower purity (23–61%) showed MICs of 4–16 mg/L. Once again, the activity was not dependent on the isoforms composition (batch) and/or purity of the sample.

**TABLE 4:** MICs obtained for lipopeptides against filamentous fungi after 7 days of growth in RPMI-1640 liquid media.

Lipopeptide	Composition	Purity	*R. stolonifer*	*P. variotii*	*B. fulva*
	
	(%)	(%)	MIC (mg/L)		
Amphotericin B			0.5	0.06	0.06
Surfactin (S)		92	>64	>64	>64
Fengycin (F)		84	>64	>64	>64
Mycosubtilin (M-I)		81	>64	2	2
Surfactin/Fengycin (S/F)	46:54	72	>64	>64	>64
Mycosubtilin/Surfactin (M/S-I)	80:20	>80	>64	2	2
Mycosubtilin III (M-III)		>80	>64	2	2
Mycosubtilin IV (M-IV)		89	>64	2	2
Mycosubtilin/surfactin II (M/S-II)	80:20	>80	>64	1	1
Mycosubtilin/surfactin III (M/S-III)	80:20	61	>64	4	4
Mycosubtilin/surfactin (M/S-23%)	80:20	23	>64	8–16	8–16
Mycosubtilin/surfactin (M/S-34%)	80:20	34	>64	8–16	8–16
Mycosubtilin/surfactin (M/S-42%)	80:20	42	>64	8	8

This is the first report on the effect of lipopeptides on the growth of *P. variotii* and *B. fulva*, with results being similar to those reported in the literature for other fungal strains. For example, when mycobubtilin and M/S (50% w/w) were tested against *Fusarium oxysporum* MICs were 10 and 5 mg/L, respectively, while surfactin showed no inhibition at concentrations up to 500 mg/L (Mihalache et al., 2017). In another study, half maxima inhibitory concentrations for mycosubtilin and M/S (50% w/w) against *Zymoseptoria tritici* were 1.4 and 4.5 mg/L, respectively (Mejri et al., 2017).

### Antimicrobial Activity of Nisin-Lipopeptides Combinations

Since lipopeptides did not clearly demonstrate an effect on the growth of bacteria, the combination of nisin with the active lipopeptides was examined against *C. divergens* and filamentous fungi.

**FIGURE 2:**
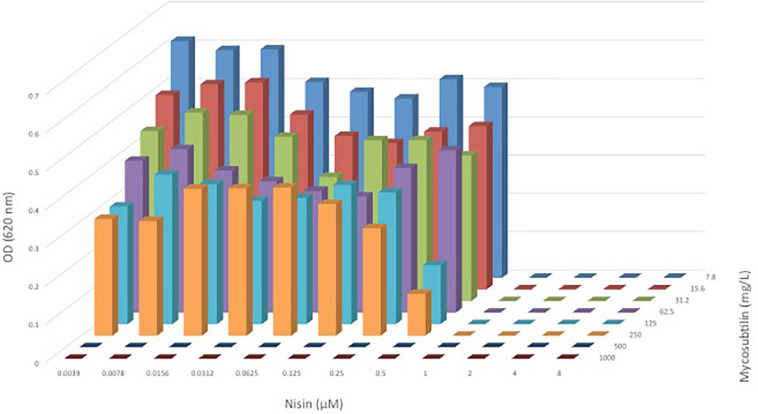
OD_620nm_ of *C. divergens* after 24 h of growth in a broth microdilution checkerboard panel containing nisin (0.039–8 μM or) and mycosubtilin (M-I, 7.8–1,000 mg/L).

A shown in Figure 2, the combination of nisin with mycosubtilin (M-I) had no effect on the MICs obtained for each individual compound, suggesting no synergistic nor antagonistic effects. It must be noted that the mycosubtilin sample tested here (M-I) showed antimicrobial activity (MIC = 500 mg/L) whereas other samples did not (see above), as explained above, this may be due to other antimicrobials in the sample which have not been identified. In any case, these results show that combining nisin with mycosubtilin did not affect the antimicrobial activity of nisin which was very effective against *C. divergens* (MIC = 1 mM = 3.35 mg/L). These results were confirmed when mixing 10 mg/L nisin with mycosubtilin (M-IV) at same range of concentrations as in Figure 2. Mixing of nisin at 10 mg/L with mycosubtiliin up to 500 mg/L led to complete inhibition of bacterial growth (data not shown). The same was observed when nisin was mixed with the mycosubtilin:surfactin mixture (M/S-III).

When nisin was combined with surfactin, at the same range of concentrations as mycosubtiliin, neither synergistic nor antagonistic effects were observed as the MIC obtained for *C. divergens* was the same as for nisin alone (3.35 mg/L), regardless of the surfactin concentrations (data not shown).

The same mycosubtlin (M-IV) and M/S (M/S-III) samples were combined with nisin (Table 5) to test antifungal activity. Nisin alone showed no inhibition of *P. variotii* and *B. fulva* at concentrations up to 125 mg/L. Furthermore when combined with mycosubtilin or MS at a fixed concentration (10 mg/L) the MIC values obtained were the same as those for mycosubtilin or mycosubtilin:surfactin mixture alone (Table 5). Thus, no synergistic or antagonistic effect was conferred by nisin.

**TABLE 5:** Antifungal activities of nisin and combinations with lipopeptides.

Compound(s)	*C. krusei*	*R. stolonifer*	*P. variotii*	*B. fulva*
	
	Lipopeptide MIC (mg/L)			
Nisin,	>125	>125	>125	>125
Nisin + M-IV	16	>64	2	2
Nisin + M/S-III	64	>64	4	4
M-IV	16	>64	2	2
M/S-III	64	>64	4	4

*MICs (mg/L) of nisin, lipopeptides alone and combinations with nisin at a fixed concentration of 10 mg/L).*

These are very interesting results which demonstrate that by combining nisin with mycosubtiliin or M/S a combined antimicrobial activity can be achieved which broadens the spectra of action, i.e., activity against both Gram-positive bacteria (susceptible to nisin) and fungi (susceptible to M and M/S).

### Cytotoxicity of Lipopeptides Produced by *B. subtilis*

**FIGURE 3:**
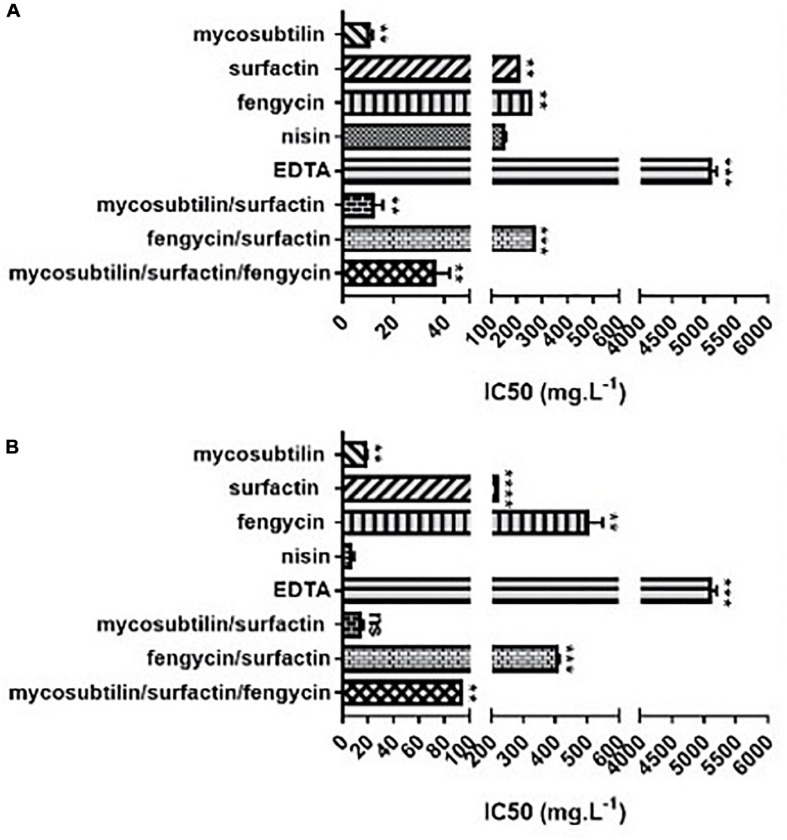
*In vitro* cytotoxicity of different lipopeptides after 48 h of incubation and representation of their half-maximal inhibitory concentration (IC_50_) against **(A)** Vero-SF cells and **(B)** Caco-2 cells. Data are shown as mean ± SD. *p < 0.05, **p < 0.01, ***p < 0.001, and ****p < 0.0001.

**FIGURE 4:**
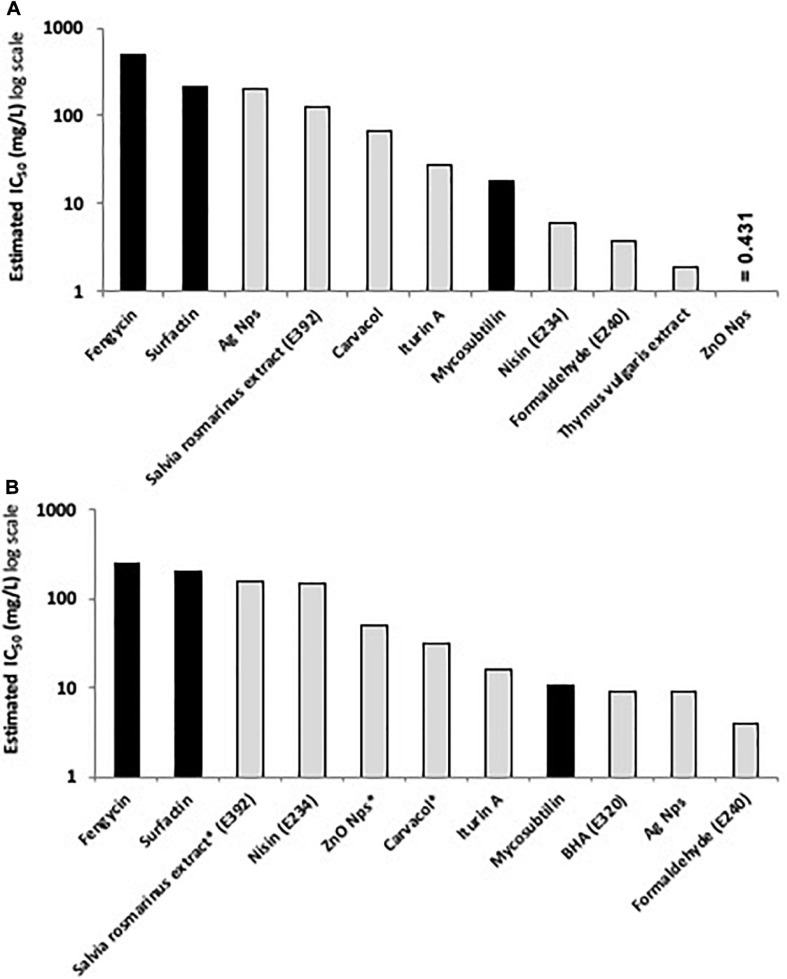
Comparative histograms presenting the estimated IC_50_ (in mg/L) of the tested lipopeptides (black columns) compared to the IC_50_ of other active preservatives/antioxidant molecules (light gray columns) using MTT assay on **(A)** Caco-2 cells and **(B)** Vero cells after 24 h of contact, except for molecules with asterisks where the contact time was 48 h (data originated from EFSA database and literature review).

According to the results obtained, lipopeptides showed different levels of cytotoxicity. To put into perspective, the lipopeptide’s values have been experimentally compared against the food preservatives nisin (E234) and EDTA (E386) which are authorized in Europe by EFSA and graded as GRAS by the FDA. As shown in Figure 3, EDTA (E386) showed no cytotoxicity towards the two cell lines tested, and always exhibited the highest IC_50_. Mycosubtilin was the lipopeptide with the lowest IC_50_ on the two cell lines (between 10 and 20 mg/L), but it was three times less cytotoxic than the food additive nisin (E234) on Caco-2 cells (Figure 3B). Moreover, the different lipopeptides, alone or as a mixture, were less cytotoxic on Caco-2 cells compared to the food grade additive nisin (E234) (Figure 3B). However, toxicity against Vero cells showed that only fengycin, surfactin and their mix were less cytotoxic than nisin (E234) (Figure 3A). Moreover, it is important to highlight that the IC_50_ values of mycosubtilin and mycosubtilin/surfactin mixtures were largely above their MIC against filamentous fungi (1–2 mg/L) (Table 4). In a similar fashion, nisin’s IC_50_ (even against Caco-2 cells) was above its MIC against Gram-positive bacteria (Table 1). The estimated IC_50_ of the lipopeptides and nisin (E386) determined in these experiments (cytotoxicity MTT assays on undifferentiated Caco-2 and Vero cells with 24 h of contact) were then compared to already published IC_50_ values (using a similar protocol to ours) of others preservatives/antioxidants food additive molecules or food packaging additive molecules. The choice of these molecules was made based on available data and by taking representative molecules from each category of food additives or food packaging additive molecules (antimicrobial peptides, synthetic antioxidant, chemical preservative, plant extract and essential oil, and nanoparticles) (Figure 4). Nanoparticles have drawn recently great interest due to their various properties (barrier properties, antimicrobial properties, etc…) that make them advantageous in food and food packaging applications (Bumbudsanpharoke et al., 2015), that’s why it seemed necessary to add them here. Data of these preservatives/antioxidants such as formaldehyde (E240) (Marcsek et al., 2007; Boncler et al., 2019), *Salvia rosmarinus* extract (E392) (Aherne et al., 2007; Waller et al., 2016), butylhydroxyanisol (BHA) (E320) (Labrador et al., 2007) and biological extracts such as carvacol (the main component of many essential oil) (Llana-Ruiz-Cabello et al., 2014; Mooyottu et al., 2014), iturin A (another antifungal lipopeptide produced by *B. subtilis*) (EFSA Panel on Additives and Products or Substances used in Animal Feed [FEEDAP], 2017; Zhao et al., 2018), *Thymus vulgaris* oil extract (Pérez-González et al., 2019) and nanoparticles such as ZnO NP (Song et al., 2014; Venkateasan et al., 2017) and Ag NP (Song et al., 2014; Kumar et al., 2017) have been collected from literature. In conclusion, as shown in Figure 4, lipopeptides had low toxicity on human cell lines. In fact, the *in-vitro* test against these cell lines simulated the detoxification by the kidney (Vero SF cells), as well as ingestion and absorption by the intestine (Caco-2 cells). It has to be noted that few reports, published recently, have studied the IC_50_ of lipopeptides in this context. In these studies, the IC_50_ of iturin A was estimated between 28 mg/L (Zhao et al., 2019) and 42 mg/L (Zhao et al., 2018) on Caco-2 cells around 16 mg/L on Vero cells (EFSA Panel on Additives and Products or Substances used in Animal Feed [FEEDAP], 2017) which corresponded to the range of values obtained with mycosubtilin in this study. When we compare lipopeptides and in particular, mycosubtilin with other molecules used in the food industry with antimicrobial and/or antioxidant activity we observe a very acceptable cytotoxicological profile. According to the results on these two cell lines it is possible to distinguish three groups: For Caco cells, group 1 of molecules with low cytotoxicity, IC_50_ above 100 mg/L (fengycin, surfactin, Ag nanoparticules, rosemary extract (E392)), group 2 with moderate cytotoxicity with IC_50_s between 10 and 100 mg/L (carvacol, iturin A, and mycosubtilin) and finally group three with significant cytotoxicity with IC_50_ lower than 10 mg/L (nisin (E234), formaldehyde (E240), thyme extract, ZnO nanoparticles); for Vero cells, group 1 (fengycin, surfactin, rosemary extract (E392), and nisin (E234)), group 2 (ZnO nanoparticles, carvacol, iturin A, and mycosubtilin); group 3 (BHA (E320), Ag nanoparticles, and formaldehyde (E240)). Overall these results are encouraging, and although further tests will need to be carried out, they suggest that lipopeptides can be safe to use in food applications such as food packaging at the concentrations that they exhibit antifungal activity.

## Conclusion

Lipopeptides had no antimicrobial activity against the food spoilage bacteria tested. Some samples of mycosubtilin and M/S mixture from the same two batches showed activity, particularly against *B. thermospacta* however, this activity was not reproduced with samples from other batches; most likely, as we demonstrated in this work, the activity could be attributed to other antimicrobials produced by *B. subtilis* such as, subtilin. Mycosubtilin and M/S samples showed for the first time strong antifungal activity, against food-related fungi despite their isoform composition and purity, with MICs of 1–16 mg/L. Interestingly they were not toxic at those concentrations when tested on a range of human cell lines here for the first time. Furthermore, these lipopeptides present cytotoxicity comparable to already marketed food additives suggesting their possible novel use in food applications. Finally, combinations of nisin and mycosubtilin or nisin and mycosubtilin/surfactin were found to be active against both bacteria and fungi with activities similar to those of the individual components. These are very interesting and novel results which can lead to the development of combined antimicrobials with broader spectrum of action and low toxicity showing potential as new food additives.

## Data Availability Statement

The raw data supporting the conclusions of this article will be made available by the authors, without undue reservation.

## Author Contributions

KK: methodology, investigation, validation, writing-original draft, writing-review and editing, and visualization. NM: formal analysis, investigation, writing/reviewing, and data curation. KAK: conceptualization, resources, writing original draft and editing, supervision, resources, and funding. PJ: conceptualization, resources, writing original draft and editing, reviewing, supervision, resources, project administration, and funding. FC: conceptualization, resources, writing original draft and editing, reviewing, supervision, project administration, validation, and funding. XG: conceptualization, development or design of methodology, and writing/reviewing. RR: supervision, writing/reviewing, and validation. CD: investigation and project administration. GV: investigation, methodology, and validation. All authors contributed to the article and approved the submitted version.

## Conflict of Interest

CD and GV were employed by the company Lipofabrik. FC is one of the cofounders of the Lipofabrik company. The remaining authors declare that the research was conducted in the absence of any commercial or financial relationships that could be construed as a potential conflict of interest.
