# Supplementary material for: Antimicrobial Activity of Lipopeptide Biosurfactants Against Foodborne Pathogen and Food Spoilage Microorganisms and Their Cytotoxicity
Source: Front Microbiol. 2021 Jan 11;11:561060. doi: 10.3389/fmicb.2020.561060 (PMC7829355; doi:10.3389/fmicb.2020.561060)

Table S1. Relative abundance of mycosubtilin and M/S samples used in the present study.

Lipopeptide	M-I	M-II	M-III	M-IV	M/S-I	M/S-II	M/S-III	M/S-23%	M/S-34%	M/S-42%
Mycosubtilin isoforms										
C_{15}	6.3	8.3	3.4	3.5	3.6	6.2	1.5	1.8	1.7	0.7
C_{16}	28.1	6.3	40.1	39.8	36.6	10.5	40.5	28.5	27.3	27.5
C_{17}	50.5	78.1	52.4	51.7	48.2	74.1	53.1	62.9	63.7	64.2
C_{18}	15.1	7.3	4.1	5.0	11.6	9.2	4.9	6.8	7.3	7.6
Total	100	100	100	100	100	100	100	100	100	100
Surfactin isoforms										
C_{12}					21.5	21.4	26.9	13.8	15.6	18.0
C_{13}					37.1	35.9	28.4	17.0	17.8	16.4
C_{14}					22.7	15.6	26.4	15.7	17.1	26.0
C_{15}					16.5	18.4	14.1	29.5	28.5	23.8
C_{16}					2.2	7.7	4.2	24.0	21.0	15.6
Total					100	100	100	100	100	100

Table S2. MICs of lipopeptides against *C. krusei* after 24 h and 48 h growth in RPMI-1640 liquid media

**Lipopeptide**	**Composition (%)**	**Purity (%)**	**MIC (mg/L)**	
			24h	***48h***
Amphotericin B			0.5	0.5
Surfactin (S)		92	> 256	> 256
Fengycin (F)		84	> 256	> 256
Mycosubtilin (M-I)		81	16	64
Surfactin/Fengysin (S/F)	46:54	72	> 256	> 256
Mycosubtilin/Surfactin (M/S-I)	80:20	> 80	32	32 – 64
Surfactin/Mycosubtilin/Fengycin (S/M/F)	30.7:32.4:36.9	80	32	64
Mycosubtilin III (M-III)		> 80	16	16
Mycosubtilin IV (M-IV)		89	16	16
Mycosubtilin/surfactin II (M/S-II)	80:20	> 80	32 – 64	32 – 64
Mycosubtilin/surfactin III (M/S-III)		61	64	64
Mycosubtilin/surfactin (M/S-23%)		23	32	32
Mycosubtilin/surfactin (M/S-34%)		34	16	16
Mycosubtilin/surfactin (M/S-42%)		42	32	32

Figure S1. Structure of lipopeptides families (a) Surfactin, (b) Plipastatin (fengycin), (c) Mycosubtilin.

(a)

(b)

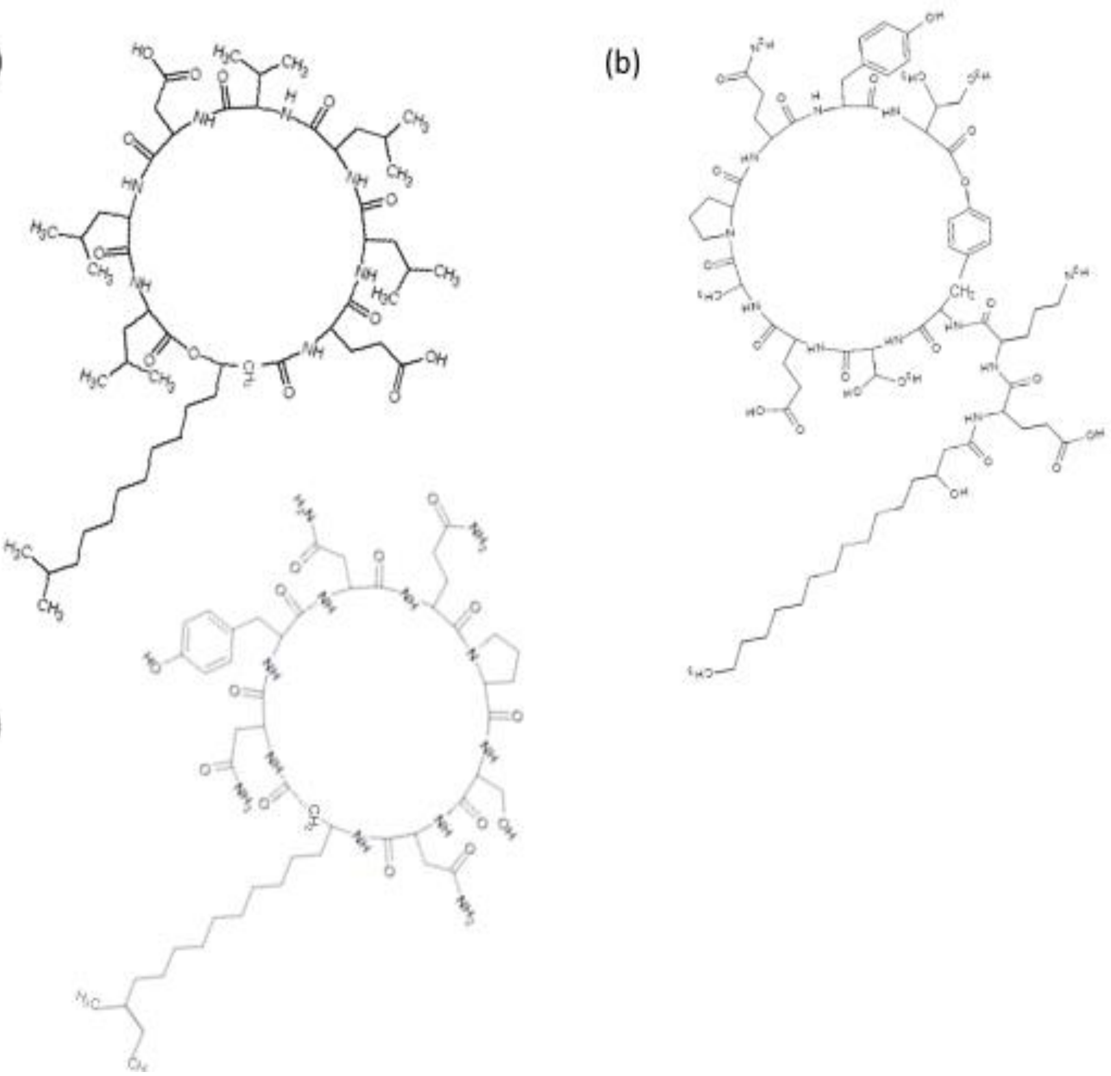

(c)

Fig S2: HRMS analysis of batch sample M-I. The peak at m/z =1107 corresponds to mycosubtilin C17 and the peak at m/z= 830 corresponds to quadricharged ions of subtilin

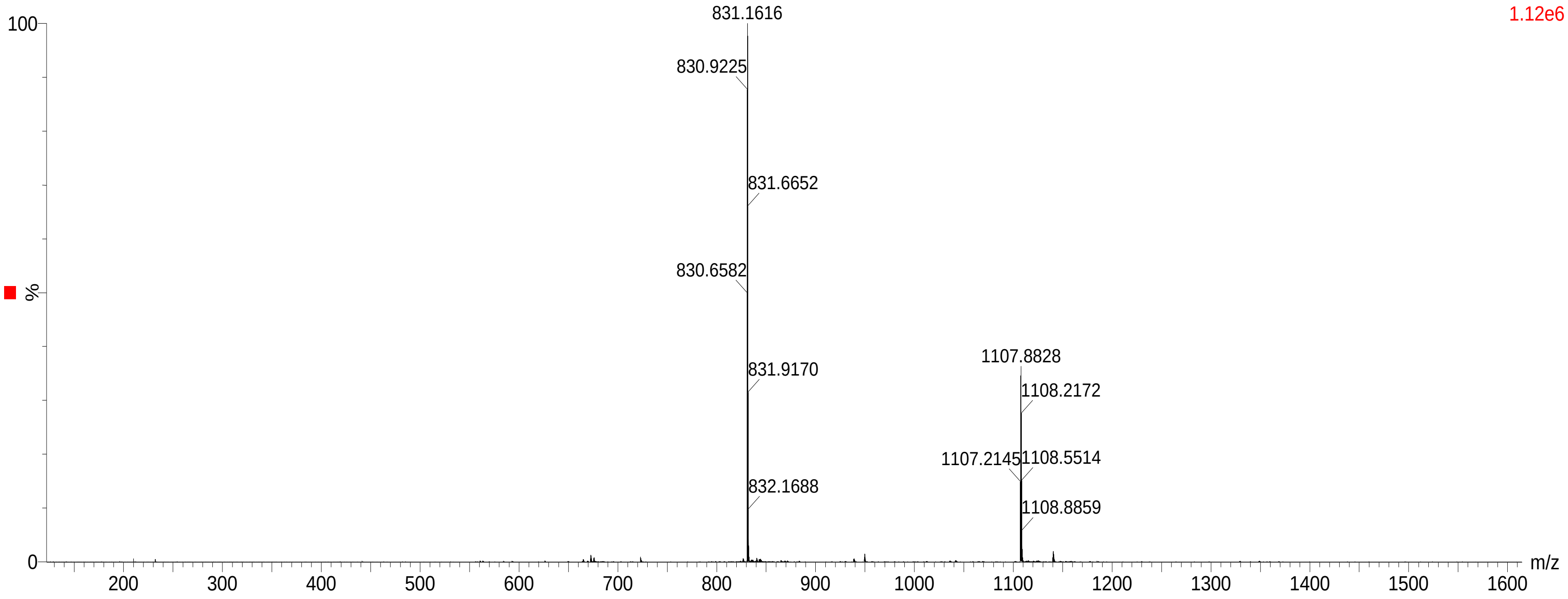

Supplement: Supplementary file 1 [file Data_Sheet_1.pdf]
